# CyberKnife Versus Four-Dimensional Computed Tomography-Guided Stereotactic Body Radiation Therapy in the Treatment of Lung Cancer: A Case Report

**DOI:** 10.7759/cureus.82138

**Published:** 2025-04-12

**Authors:** Lingrong Tang, Lei Yao, Guang Li

**Affiliations:** 1 Department of Radiotherapy, The First Hospital of China Medical University, Shenyang, CHN

**Keywords:** 4-dimensional, cyberknife, lung cancer, real-time tumor tracking, stereotactic body radiation therapy

## Abstract

Stereotactic body radiation therapy (SBRT) has emerged as a critical therapeutic option for treating lung and other solid tumors. Two prominent high-precision SBRT techniques currently in use are four-dimensional computed tomography-guided linear accelerator-based SBRT (4DCT-SBRT) and CyberKnife. This case presents a patient diagnosed with two distinct pulmonary lesions, each treated separately using 4DCT-SBRT and CyberKnife. By comparing target delineation, dose distribution, lesion response, and the capability to spare normal tissues, we evaluate the specific advantages of CyberKnife for particular clinical scenarios.

## Introduction

Precision radiotherapy is a pivotal component of modern oncology, delivering high-dose radiation accurately to tumor sites in a brief treatment period while minimizing damage to adjacent normal tissues. Stereotactic body radiation therapy (SBRT) has emerged as a critical therapeutic option for treating inoperable stage I non-small cell lung cancer and other solid tumors [1,2], offering outcomes comparable to surgery, although current evidence remains conflicting regarding optimal patient selection between SBRT and surgical intervention [3,4]. Leading modalities of SBRT include 4DCT-SBRT, CyberKnife, proton SBRT, MR-guided SBRT, and helical tomotherapy [5-9]. Among these techniques, four-dimensional computed tomography-guided linear accelerator-based SBRT (4DCT-SBRT) and CyberKnife are more frequently used in clinical practice.

4DCT-SBRT utilizes four-dimensional computed tomography (4DCT) to capture respiratory-induced tumor motion, improving treatment accuracy. Techniques like respiratory gating and internal target volume (ITV) expansion effectively manage tumor movements, making this modality ideal for a broad range of solid tumors in organs such as the lungs, liver, and pancreas [2,10-12].

CyberKnife integrates a robotically controlled linear accelerator combined with Synchrony Respiratory Tracking technology. This approach tracks fiducials placed in the tumor in real-time without additional gating or ITV expansions, allowing for smaller target volumes and enhanced protection of adjacent normal tissues [13,14].

In this case report, we analyze a patient treated with 4DCT-SBRT in 2020 and subsequently with CyberKnife in 2024, highlighting these techniques' clinical distinctions and comparative advantages.

## Case presentation

A 70-year-old male patient presented in September 2020 with a chronic cough. Computed tomography (CT) imaging identified a solid lesion in the left lower lung lobe, which was subsequently confirmed as adenocarcinoma via CT-guided biopsy. The lesion tested negative for mutations in EGFR, ALK, ROS1, BRAF, MET, and RET. Following a comprehensive clinical evaluation, the patient was diagnosed with early-stage lung cancer, classified as T1bN0M0, stage IA2, according to the American Joint Committee on Cancer (AJCC) 8th edition. Due to underlying conditions including cerebral thrombosis and coronary artery disease, the patient declined surgical intervention. Consequently, treatment using 4DCT-SBRT was initiated on an Elekta Versa HD linear accelerator.

**Figure 1:**
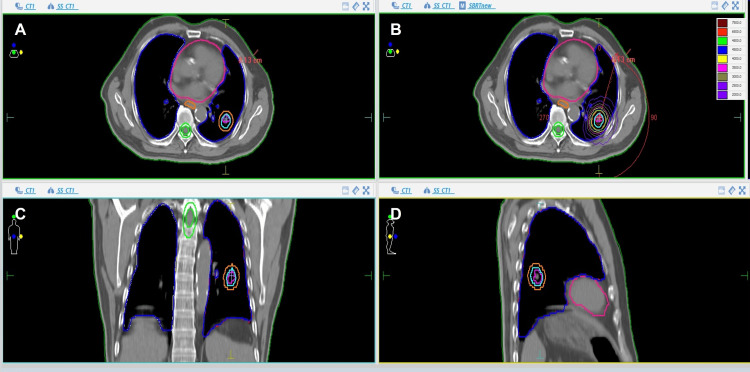
Target delineation and radiotherapy plan of 4DCT-SBRT (A, C, D) Axial, coronal, and sagittal views showing target volume delineation. The blue line indicates ITV, and the orange line indicates PTV. (B) VMAT treatment plan. ITV: Internal target volume; PTV: Planning target volume; VMAT: Volumetric modulated arc therapy; 4DCT-SBRT: Four-dimensional computed tomography-guided linear accelerator-based stereotactic body radiation therapy

Planning involved respiratory-gated 4DCT imaging with the ITV method. The gross tumor volume (GTV) was delineated on lung window settings (window width: 1600 HU; window level: -600 HU), and the ITV encompassed the combined trajectory of the GTV across all respiratory phases. The clinical target volume (CTV) was equivalent to the GTV without additional margins, while the planning target volume (PTV) was determined by expanding the ITV by a uniform 5 mm margin. A total dose of 50 Gy was delivered in five fractions over two weeks, each lasting approximately 10 minutes (Figure 1).

**Figure 2:**
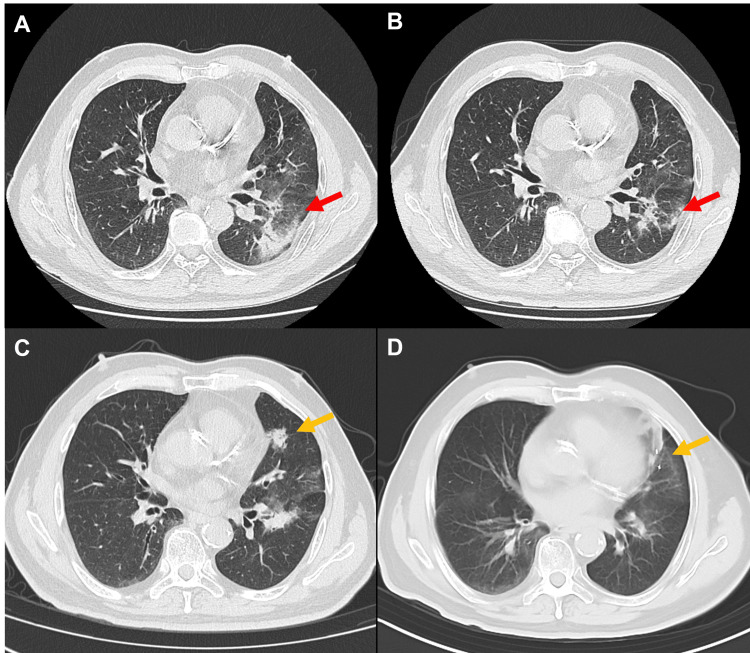
Radiographic images illustrating radiation pneumonitis and tumor response (A) Axial CT image showing grade 1 radiation pneumonitis at eight months post-treatment (red arrow). (B) Axial CT image demonstrating gradual resolution of radiation pneumonitis at 15 months post-treatment (red arrow). (C) Axial CT scan obtained in January 2024 showed a newly detected lesion in the left upper lobe (orange arrow). (D) Axial CT image demonstrating significant tumor regression at 12 months after treatment (orange arrow). CT: Computed tomography

Treatment resulted in a partial tumor response; however, mild radiation pneumonitis (Common Terminology Criteria for Adverse Events (CTCAE) version 5.0, grade 1) appeared eight months post-treatment, which gradually resolved (Figures 2A, 2B). A new enlarging lesion in the left upper lung lobe was identified during a routine follow-up at another medical facility. Enhanced CT scans demonstrated marked enhancement suggestive of malignancy (Figure 2C). Due to the lesion's proximity to the heart, biopsy was deemed high-risk and thus avoided. Whole-body imaging confirmed the absence of other malignant lymph nodes or distant lesions.

**Figure 3:**
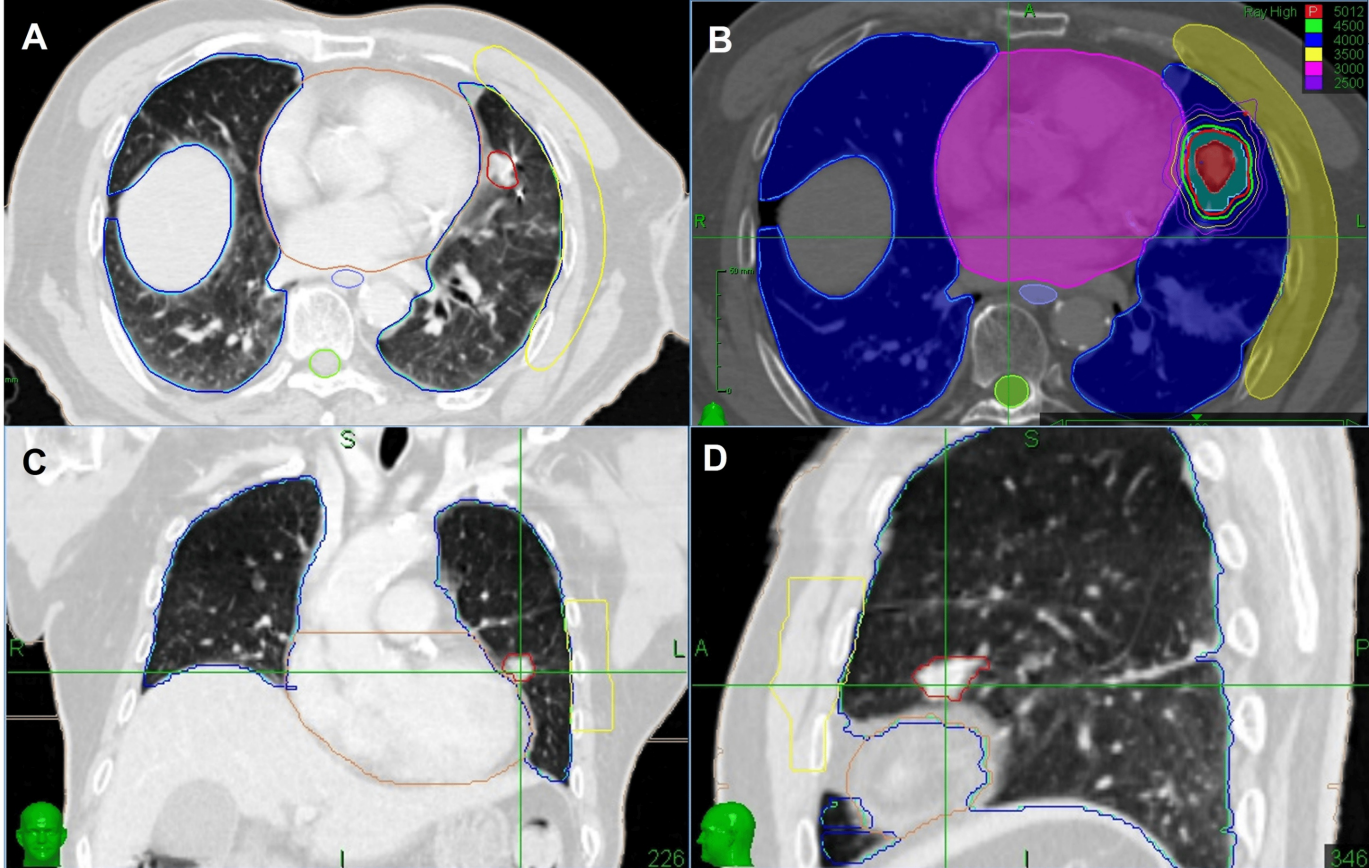
Target delineation and radiotherapy plan of CyberKnife (A, C, D) Axial, coronal, and sagittal views showing target volume delineation. The red line indicates the GTV. (B) CyberKnife treatment plan. GTV: Gross tumor volume

In February 2024, this lesion was treated using CyberKnife SBRT on the Accuray CyberKnife M6 system. The treatment utilized Synchrony Respiratory Tracking technology with fiducial markers, eliminating the need for ITV-based planning. The simulation was performed using contrast-enhanced CT imaging with a slice thickness of 1.5 mm, acquired during end-expiratory breath-hold. The GTV was delineated on lung window settings (window width: 1600 HU; window level: -600 HU), and the PTV was defined as the GTV expanded uniformly by a 5 mm margin. The lesion was treated with a dose of 50 Gy in five fractions, which was delivered over two weeks, with each fraction lasting approximately 30 minutes (Figure 3). This treatment also achieved a partial tumor response; notably, no side effects were observed (Figure 2D).

## Discussion

Comparing precision and target volume

Respiratory-induced tumor movements pose challenges in lung cancer radiotherapy, typically managed either by restricting tumor mobility such as abdominal compression, deep inspiration breath-hold, active breathing control, or expanding the target volume via ITV strategies [14,15]. The former is difficult for elderly and weak patients to cooperate with, while ITV strategies may increase irradiated lung volumes, consequently elevating radiation-induced side effects.

4DCT-SBRT relies on ITV-based planning, which is derived from all phases of respiratory motion. While this approach effectively covers tumor movement, it requires larger margins around the tumor, potentially increasing the volume of normal tissue irradiated [12]. Consequently, this could elevate the risk of pneumonitis or fibrosis, particularly in cases of lesions close to critical structures, such as the heart in this case.

CyberKnife differs from 4DCT-SBRT in its approach to sparing normal tissue. The CyberKnife system employs real-time respiratory tracking technologies, such as the fiducial, X-sight lung tracking, and synchrony respiratory tracking system, allowing continuous adjustment of the radiation beam to follow tumor motion during respiration accurately [16-18]. This technology facilitates highly accurate radiotherapy without needing target volume expansions, minimizing radiation exposure to adjacent healthy tissues and critical structures [18,19].

Treatment duration and patient tolerance

Conventional SBRT was performed using 8-12 non-coplanar static beams, and treatment delivery generally took more than 20min. However, through VMAT, the delivery time was much shorter (approximately 10 minutes), ensuring better patient tolerance [20].

Studies have demonstrated that CyberKnife lung SBRT is safe and achieves progression-free survival and overall survival comparable to conventional SBRT [13,19,21,22]. The study by Diamant et al. demonstrated that CyberKnife lung SBRT achieved superior distant metastasis-free survival compared with VMAT [5]. Multiple dosimetric studies comparing CyberKnife with conventional linear accelerator-based SBRT plans have indicated that both modalities achieve clinical dosimetric goals; however, CyberKnife plans typically deliver higher monitor units (MUs) but produce lower doses at 2 cm from the target (D2cm) [23-25]. Consequently, CyberKnife treatments require longer delivery times (approximately 30 minutes per fraction) but offer superior sparing of organs at risk (OARs). Clinically, this translates into reduced radiation-induced toxicity and potentially enhanced patient quality of life.

It is important to note that CyberKnife treatment for lung SBRT necessitates continuous respiratory tracking, and circumstances such as coughing may require recalibration, thereby prolonging the treatment duration and potentially impacting patient tolerance.

Moreover, CyberKnife treatments usually require pre-treatment fiducial marker implantation to achieve precision tracking. This invasive procedure is associated with risks such as pneumothorax or bleeding, complications that do not occur with 4DCT-SBRT [17].

Clinical recommendations

CyberKnife and 4DCT-SBRT are both suitable for lung SBRT. CyberKnife is better suited to treat highly mobile tumors (e.g., lower lobe lung, liver, pancreatic tumors), leveraging its precise real-time tracking capabilities to offer superior sparing of OARs.

4DCT-SBRT is particularly suitable for pulmonary tumors with minimal movement or high fiducial marker implantation-associated risk, and for elderly or weak patients unable to stand prolonged treatment duration.

## Conclusions

This case study demonstrates that both 4DCT-SBRT and CyberKnife effectively treat pulmonary tumors. CyberKnife, however, provides superior precision and improved sparing of normal tissues due to its smaller target volumes. Personalized choice of radiotherapy modality should consider patient health status, tumor characteristics, and proximity to critical structures to optimize outcomes and minimize side effects.
